# Expression Pattern of AIFM3, VGLL4, and WNT4 in Patients with Different Stages of Colorectal Cancer

**DOI:** 10.3390/cancers17020166

**Published:** 2025-01-07

**Authors:** Danijel Bevanda, Anita Racetin, Nela Kelam, Natalija Filipović, Mateo Bevanda, Marina Rudan Dimlić, Jelena Budimir, Daniela Bevanda Glibo, Ivana Bevanda, Danica Ramljak, Katarina Vukojević

**Affiliations:** 1 Department of Gastroenterology, School of Medicine, University of Mostar, University Hospital Mostar, Bijeli Brijeg bb, 88000 Mostar, Bosnia and Herzegovina; danijel.bevanda@gmail.com (D.B.); ela.bevanda@gmail.com (D.B.G.); 2 Department of Anatomy, Histology and Embryology, Laboratory for Early Human Development, University of Split School of Medicine, Šoltanska 2A, 21000 Split, Croatia; anita.racetin@mefst.hr (A.R.); nela.kelam@mefst.hr (N.K.); natalija.filipovic@mefst.hr (N.F.); 3 Department of Surgery, School of Medicine, University of Mostar, University Hospital Mostar, Bijeli Brijeg bb, 88000 Mostar, Bosnia and Herzegovina; bevanda333@gmail.com; 4 Mediterranean Institute for Life Sciences (MedILS), University of Split, Meštrovićevo Šetalište 45, 21000 Split, Croatia; mrdimlic@medils.unist.hr (M.R.D.); jbudimir@medils.unist.hr (J.B.); dramljak@medils.unist.hr (D.R.); 5 Department of Endocrinology, School of Medicine, University of Mostar, University Hospital Mostar, Bijeli Brijeg bb, 88000 Mostar, Bosnia and Herzegovina; bjelanovic.ivanaaa@yahoo.com; 6 Center for Translational Research in Biomedicine, University of Split School of Medicine, Šoltanska 2A, 21000 Split, Croatia

**Keywords:** colorectal cancer, AIFM3, VGLL4, WNT4

## Abstract

Background/Objectives: Colorectal cancer (CRC) remains a significant health burden, and its delayed diagnosis at advanced stages leads to poor survival outcome. Detection of known and novel prognostic markers is essential. In this study, the status of likely prognostic markers—the apoptotic inducing factor (AIFM3), vestigial-like family member 4 (VGLL4), and WNT4—was evaluated. Methods: AIFM3, VGLL4, and WNT4 expression in CRC tissues across different stages (Dukes A–D) were analyzed using histological immunofluorescence staining and RNA sequencing analyses. Results: In advanced CRC stages, progressive loss of normal crypt architecture, reduction of goblet cells, and necrotic debris were detected along with differential expression patterns of AIFM3, VGLL4, and WNT4. AIFM3 exhibited high reactivity in the lamina propria of healthy tissue and Dukes A, but this was diminished in advanced CRC stages. VGLL4 expression, initially confined to the lamina propria, increased significantly in the epithelium of Dukes B and C, with a cytoplasmic localization pattern. WNT4 expression was elevated in the CRC epithelium across all stages, contrasting with a significant reduction in lamina propria reactivity. RNA sequencing corroborated these findings, showing significant downregulation of AIFM3 and WNT4 and upregulation of VGLL4 in CRC tissues compared to controls. Expression of AIFM3 and WNT4 showed no correlation with survival outcome, while low VGLL4 expression was correlated with better survival outcome. Conclusions: The results suggest distinct roles for AIFM3, VGLL4, and WNT4 in CRC progression, highlighting only VGLL4 as a potential prognostic marker. Further evaluation of VGLL4 and its specific role in CRC progression remains to be elucidated.

## Simple Summary

This research considers means of improving the early detection and treatment of colorectal cancer (CRC), a common and deadly form of cancer often identified too late for effective treatment. In our study, we examined AIFM3, VGLL4, and WNT4 in cancerous and healthy tissues at various stages of CRC. We found that these markers behave differently as the cancer advances. AIFM3 appears in healthy tissue and early cancer stages but disappears as the cancer worsens. VGLL4 increases in the affected tissues as the cancer progresses, particularly noticeable from early to more advanced stages. WNT4 is higher in cancerous tissues, but decreases in the supportive tissue surrounding cancer cells as the disease advances. Low VGLL4 levels are linked to better patient survival, unlike the other two markers. This finding suggests that VGLL4 could be useful as an indicator of CRC prognosis, potentially guiding treatment approaches.

## 1. Introduction

Cancer exerts tremendous financial strain on communities and health systems. As the second leading cause of death worldwide, it accounts for approximately 10 million deaths a year. Cancer incidence increases with age, and it is expected that these numbers will only rise as a consequence of the growing population aging [1]. Colorectal cancer (CRC) is the third most common cancer type worldwide, accounting for approximately 10% of all cancer-related deaths [2,3]. At early stages, CRC is often symptomless, and it is usually diagnosed in an advanced and/or metastatic stage when treatment options are limited. Treatment success is influenced by the cancer genotype and the corresponding tumor microenvironment [4]. There are different CRC subtypes which are classified based on unique morphological and molecular traits. Adenocarcinomas account for about 95% of colorectal cancer cases, while neuroendocrine tumors and small-cell carcinomas represent the remaining 5% [5]. The stage of the disease detected at the time of diagnosis or after surgery generally has a significant impact on the patient’s prognosis [6,7].

The stage and the metastatic potential of CRC are classified based on the TNM or a Dukes classification [8]. Genetic instability is a major characteristic of CRC and arises through two basic processes. The most common mechanism, accounting for around 84% of instances, is chromosomal instability, defined by changes in the number and shape of chromosomes, including deletions and translocations [9]. The second is related to hypermutation and microsatellite instability (MSI) as a result of poor DNA mismatch repair (MMR), and it is observed in around 15% of sporadic CRC cases [10]. However, these traditional classifications based on tumor size, location, and the stage do not fully capture the complexity of CRC. Therefore, precise analysis of specific genetic and molecular alterations within the tumor might provide important and patient-specific information for future personalized therapy options.

The molecular status of proteins associated with cellular death and growth belonging to apoptotic, Wnt/β catenin, and Hippo YAP pathways might be associated with different stages of CRC and represent important prognostic markers and possible therapeutic targets.

Apoptotic inducing factor M3 (AIFM3), a mitochondrial protein involved in the execution phase of apoptosis [11], is widely expressed across multiple tissues and shows abnormal expression in various cancers [12]. Although AIFM3 has been found in colon tissue, until recently, clinical implications of AIFM3 were not reported for CRC.

Vestigial-like family member 4 (VGLL4) is a member of the VGLL family of transcriptional cofactors and is involved in negative regulation of the Wnt signaling pathway, negative regulation of cell growth, and negative regulation of Hippo signaling. VGLL4 levels in cancer cells are typically lower compared to normal tissues, and reduced expression of VGLL4 is often associated with poor survival outcomes in numerous cancers, including lung, gastric, breast, colorectal, bladder, pancreatic adenocarcinoma, and esophageal squamous cancer. On the other hand, higher VGLL4 expression is positively correlated with better survival rates [13]. Its interaction with TEAD transcription factors affects the Wnt/β-catenin and Hippo–YAP1 signaling pathways, which are critical for cellular development and differentiation. Due to its negative regulation of pathways involved in tumor proliferation, the VGLL4 acts as a tumor suppressor.

WNT4 is a member of the WNT protein family, important for key cellular activities such as cell growth and development [14]. Upregulation of Wnt signaling pathways is one of the main drivers of CRC development and progression. In CRC, WNT4 is significantly elevated in patient serum and originates largely from cancerous tissues, suggesting that it may serve as a potential biomarker. The WNT4 promotes CRC progression by inducing epithelial–mesenchymal transition (EMT), activating fibroblasts, and enhancing angiogenesis through the Wnt/β-catenin signaling pathway. These effects contribute to the aggressive nature of CRC and elevate its metastatic potential [15]. WNT4 has also been observed to play a role in other cancer types, particularly breast cancer, where it may contribute to aberrant cell proliferation.

**Scheme 1:**
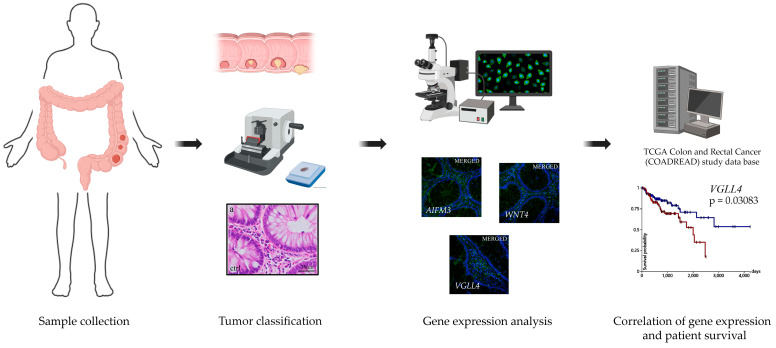
Workflow for investigating colorectal cancer gene expression and its correlation with patient survival. Samples were collected from colorectal tissue and classified based on tumor pathology. Tumor sections were analyzed for gene expression using immunofluorescence microscopy, highlighting markers such as AIFM3, WNT4, and VGLL4. Data from The Cancer Genome Atlas (TCGA) colon and rectal cancer (COADREAD) study database were used to correlate gene expression levels with patient survival outcomes.

In this study, it was hypothesized that AIFM3, VGLL4, and WNT4 proteins play an important role in molecular events leading to development of CRC and might represent an important new prognostic marker. Molecular classification of these proteins in different stages of CRC could contribute to the development of personalized treatments, particularly for individuals at higher risk Scheme 1.

## 2. Materials and Methods

### 2.1. Ethics

This study was performed in accordance with the Helsinki Declaration and has been approved by the Ethics Committee of the School of Medicine, University of Mostar.

### 2.2. Tissue Procurement and Immunohistochemistry

Patient recruitment was carried out in collaboration with healthcare professionals to identify appropriate candidates. Participants were approached directly during medical visits, followed by subsequent communication and informational sessions to guarantee informed consent. No medications were administered to participants prior to the study, as it focused on patients receiving their first CRC diagnosis. The youngest patient was 45, and the oldest was 89 years of age.

A total of 43 tumor tissue samples and control tissues were taken at laparoscopic CRC surgery in paraffin blocks (12 samples from Dukes A, 11 samples from Dukes B, 10 samples from Dukes C, and 10 samples from Dukes D) were received from the Department of Pathology, Cytology, and Forensic Medicine at University Hospital Mostar (Table 1). The study included CRC tumor samples across all cancer stages from patients of both sexes and all age ranges, as described above. Stratified randomization ensured an even distribution of cancer stages as the key characteristics across treatment groups.

Samples were prescreened, selecting only those with adequate paraffin block material for further immunohistochemistry (IHC) analysis and complete clinical data. Exclusion criteria included incomplete laboratory results, insufficient IHC material, and lack of control tissue. The control tissue was normal tissue adjacent to tumor tissue.

Tumor samples were examined and measured, with each sample containing both tumor and healthy control tissue. The tissue blocks were processed following the method outlined by Vukojevic et al. [16]. In summary, the tissues were dehydrated using an increasing series of ethanol solutions (70% to 100%) and then embedded in paraffin. Sections (4 μm) were cut from the paraffin tissue blocks by microtome (Leica RM2155, Pittsburgh, PA, USA). The sections were deparaffinized in xylene, rehydrated in decreasing ethanol solutions, and rinsed in distilled water. Histological evaluation of the collected tissue samples was performed using hematoxylin and eosin (HE) staining. The HE staining was used for assessing tissue morphology and preservation, as well as to identify tumor regions, differentiate between cancerous and normal tissues, and evaluate the histopathological features of the samples to ensure accurate classification and selection of regions for subsequent analyses.

The next step in the immunofluorescence protocol involved heating the tissue in citrate buffer (pH 6) at 95 °C in a steam bath for 15 min, then allowing it to cool to room temperature. Afterwards, the tissue sections were incubated in blocking buffer (ab64226, Abcam, Cambridge, UK) for 20 min, followed by a one-hour incubation with the appropriate primary antibody combinations (Table 2). After being rinsed in PBS, the tissue sections were incubated with the secondary antibody for one hour. After final rinsing in the PBS, sections were counterstained with 4′,6-diamidino-2-phenylindole (DAPI) and cover-slipped.

To minimize non-specific background signals, isotype-matched controls and secondary-only samples were used. Isotype-matched controls replaced the primary antibody with one of the same isotype but without specificity for the target, helping to identify non-specific binding. Secondary-only samples, where the primary antibody was omitted, allowed for the detection of any non-specific binding of the secondary antibody. These controls ensured that observed signals were specific to the target protein and not due to background noise, such as residual paraffin (Figure S1).

### 2.3. Data Collection and Processing

Hematoxylin- and eosin-stained tissue sections were analyzed by microscope (BX40, Olympus, Tokyo, Japan), and immunofluorescence images were captured by an immunofluorescence microscope (BX51, Olympus, Tokyo, Japan) mounted with a digital camera (Nikon Ri-D2, Nikon, Tokyo, Japan).

For AIFM3, VGLL4, and WNT4, positive cell counts were performed on captured immunofluorescence images for all groups, in the epithelium and lamina propria. Regarding the classification of positive cells, any cells showing nucleic, cytoplasmic, or membranous staining, regardless of intensity, were considered positive, and cells with no apparent immunofluorescent signal were considered unstained (negative), when merged with DAPI. Image processing was conducted by ImageJ (National Institutes of Health, Bethesda, MD, USA). For quantitative analysis, images were set to be divided into squares (20 μm × 20 μm at 40× magnification).

Positive and negative cell counts followed a standardized method: only squares fully encompassing the region of interest were evaluated, avoiding duplicate counts by analyzing alternate sections. Cells along the right and lower boundaries of each square were included, while those on the left and upper boundaries were excluded. The percentage of positive cells was determined by analyzing ten representative images within the regions of interest for each sample. The results were then averaged per sample and across the examined groups. Data were presented as mean ± standard deviation (SD). For image assembly, Adobe Photoshop (Adobe Photoshop CS, 2004, Berkeley, CA, USA: Peachpit Press) was applied.

### 2.4. The Examination of RNA Transcripts

The TCGA colon and rectal adenocarcinoma (COADREAD) gene expression dataset, obtained through RNA sequencing (polyA + IlluminaHiSeq), was retrieved from the Xena database at the University of California Santa Cruz (https://xenabrowser.net/datapages/; accessed on 4 May 2024) and utilized for the current studies. This dataset combined findings from TCGA colon adenocarcinoma and rectal adenocarcinoma cases, specifically those with no prior history of neoadjuvant treatment, and was measured using the Illumina HiSeq 2000 RNA Sequencing platform at the University of North Carolina’s TCGA genome characterization center. The dataset provided gene-level transcription estimates represented as log2(x + 1)-transformed RSEM normalized counts. Data regarding overall survival, gene expression of samples, and information on microsatellite instability were exported as text files and processed in Microsoft Excel 2019 MSO version 2305 (Microsoft Corp., Redmond, WA, USA). After curation of the data to remove duplicates and samples lacking expression data for the factors studied, 431 patient samples were preserved for analysis, which included 380 primary tumors (PT) and 51 normal solid tissues from healthy resection margins (STN). The expression of AIFM3, WNT4, and VGLL4 in relation to microsatellite instability (MSI) status in colorectal cancer was analyzed using a cohort of 108 patients. Among the cohort, 98 patients were classified as MSI-stable, while 10 were MSI-unstable.

### 2.5. Statistical Data Analysis

The GraphPad was used for statistical alalysis (GraphPad Software, San Diego, CA, USA). Ordinary one-way ANOVA was used, and afterwards Tukey’s multiple comparison tests was performed for protein expression comparison between control and CRC tissues (at all tumor stages) in the lamina propria and epithelium. A two-way ANOVA test was used for control tissue and each examined CRC stage within each group. Šídák’s multiple comparisons test was then used to compare the protein expression (lamina propria vs. epithelium). To compare the expression of AIFM3, VGLL4, and WNT4 markers between tumor and normal tissue specimens, an unpaired *t*-test was used. Survival analysis was conducted using expression quartiles for each gene. The statistical evaluation of survival duration was performed using the Kaplan–Meier method along with the log-rank test. The Mann–Whitney U test was used to test differences in gene expression levels between MSI-stable and MSI-unstable groups. A difference was considered statistically significant if the *p*-value was less than 0.05.

## 3. Results

### 3.1. Cellular Architecture of CRC by Hematoxylin and Eosin Staining

**Figure 1:**
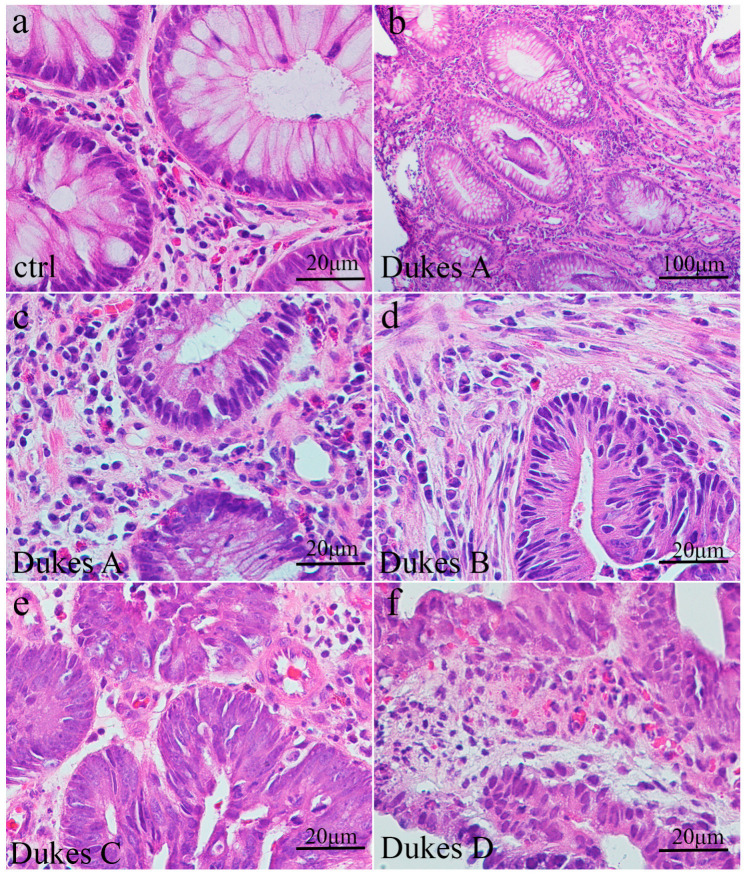
The morphology of intestinal crypts is altered in colorectal cancer tissue compared to healthy tissue. Section of healthy adult colon (**a**) with circular crypts and basally positioned nuclei within cells with underlying lamina propria; section of Dukes A human colon cancer (**b**,**c**) with moderately formed crypts lined by epithelial cells with impaired nuclear position; section of Dukes B human colon cancer (**d**) with the cellular debris within gland lumen; section of Dukes C and D (**e**,**f**) human colon with disrupted glands underlined by atypical epithelial cells.

Analysis of healthy colon tissue revealed specific structural features visualized by hematoxylin and eosin staining. Goblet cells, specialized for mucus secretion, were abundant in the epithelium that lines the inner surface of the colonic crypts (glands); these appear as clear, rounded spaces (Figure 1a). The lamina propria, a layer of connective tissue supporting the epithelium, was inter-spread between the crypts. The nuclei of columnar and goblet cells were located at the basal ends of the cells and presented with a dark purple color.

In colorectal cancer (Dukes A and B), staining reveals partially preserved crypt architecture with irregular crypt formations. Goblet cells were reduced in number, and epithelial cells exhibited nuclear abnormalities (Figure 1b–d). The section of human Dukes C and D demonstrated necrotic debris within the lumen of adenocarcinomatous glands, replaced by disorganized, malignant growth (Figure 1e,f).

### 3.2. Immunofluorescence Staining with AIFM3 Marker and Quantification of Immunoreactive Cells

**Figure 2:**
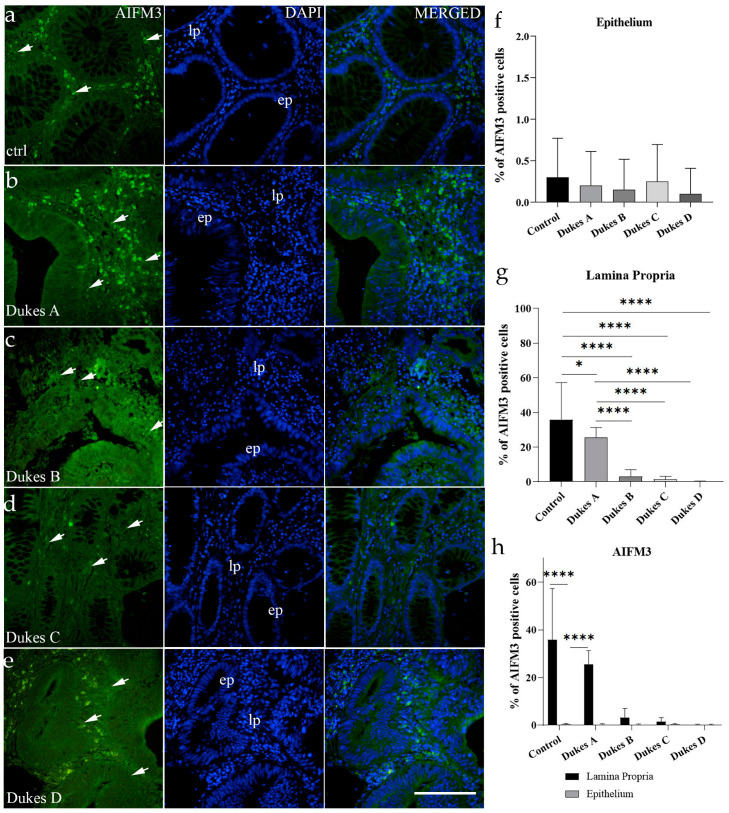
Immunofluorescence image of the control adult human colon (**a**) and CRC stages Dukes A (**b**), Dukes B (**c**), Dukes C (**d**), and Dukes D (**e**). Arrows indicate positive AIFM3 cells in the epithelium (ep) and lamina propria (lp). DAPI staining (blue nuclei). The percentages of cells positive to AIFM3 staining in control and Dukes A–D CRC stages in the epithelium (**f**) and lamina propria (**g**) were calculated from a minimum of ten representative images. For (**a**–**e**), scale bar is 100 μm. For statistical analysis, one-way ANOVA followed by Tukey’s multiple comparison tests (**f**,**g**) and two-way ANOVA test followed by Šídák’s multiple comparisons test (**h**) were used. Data are presented as the mean ± SD. Significant differences are indicated by * *p* < 0.05 and **** *p* < 0.0001.

The reactivity to AIFM3 antibody was very low in the epithelium and strong in the lamina propria in healthy colon tissue (Figure 2a). There were only 0.30% AIFM3-positive cells in the epithelium, while 35.85% AIFM3-positive cells were detected in the healthy lamina propria (Figure 2f,g). Similar to the healthy tissues, reactivity to AIFM3 in CRC Dukes A was mostly detected in the lamina propria (Figure 2b). There were 0.20% and 25.50% AIFM3-positive cells in the epithelium and lamina propria of Dukes A, respectively (Figure 2f,g).

Contrary to the healthy colon and Dukes A, more advanced stages of CRCs (Dukes B, C, and D) had low reactivity to the AIFM3 antibody in both the lamina propria and epithelium (Figure 2c–e). In Dukes B, there were 3.15% and 0.15 % AIFM-positive cells in the lamina propria and epithelium, respectively (Figure 2f,g). The CRCs with stage Dukes C had 1.45% AIFM-positive cells in the lamina propria and 0.25% in the epithelium (Figure 2f,g). AIFM-positive cells were barely detected in Dukes D cases (Figure 2e). There were only 0.10% AIFM3-positive cells in both the lamina propria and epithelium (Figure 2f,g).

The percentage of AIFM3-positive cells in the epithelium between different samples showed no significant difference in AIFM3-positive cells (*p* > 0.05) (Figure 2f). In the lamina propria, significant differences were detected between the healthy tissue and different CRC Dukes stages (Figure 2g). Significant differences were detected between Dukes A and the more advanced stages of CRC Dukes B, C, and D (*p* < 0.0001) (Figure 2g). There were no significant differences in AIFM3-positive cells between Dukes B, C, and D (*p* > 0.05).

The expression analysis of AIFM3 between the lamina propria and epithelium showed significantly higher expression in the lamina propria of the healthy tissue and Dukes A compared to the epithelium (*p* < 0.0001) (Figure 2h). There were no significant differences between lamina propria and epithelium expression in other samples (*p* > 0.05) (Figure 2h).

### 3.3. Immunofluorescence Staining with VGLL4 Marker and Quantification of Immunoreactive Cells

**Figure 3:**
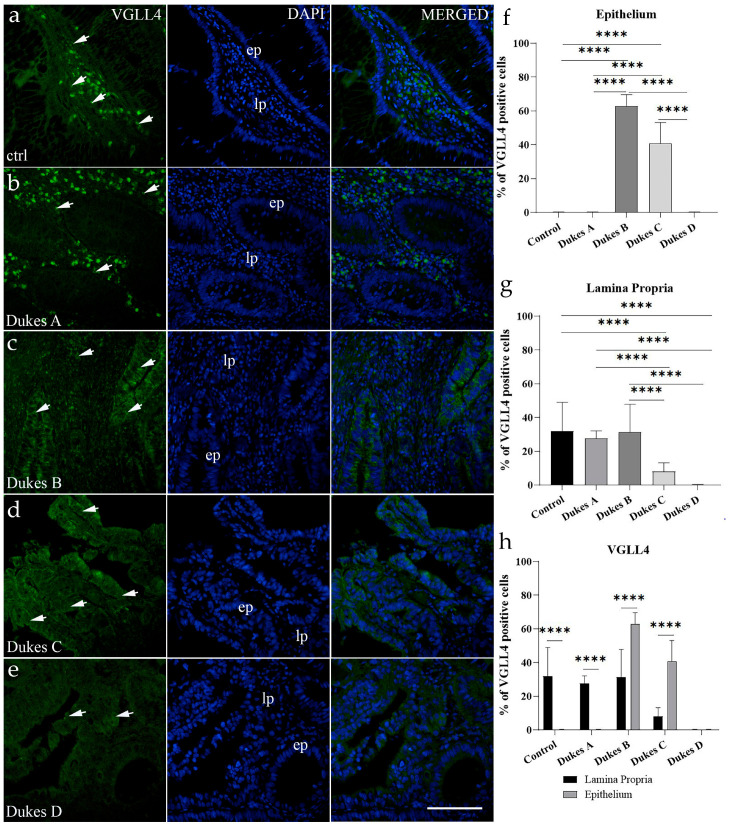
Immunofluorescence image of the control adult human colon (**a**) and CRC stages Dukes A (**b**), Dukes B (**c**), Dukes C (**d**), and Dukes D (**e**). Arrows indicate positive VGLL4 cells in the epithelium (ep) and lamina propria (lp). DAPI staining (blue nuclei). The percentages of cells positive to VGLL4 in the control and Dukes A–D CRC stages in the epithelium (**f**) and lamina propria (**g**) were calculated from a minimum of ten representative images. For (**a**–**e**), scale bar is 100 μm. For statistical analysis, one-way ANOVA followed by Tukey’s multiple comparison tests (**f**,**g**) and two-way ANOVA test followed by Šídák’s multiple comparisons test (**h**) were used. Data are presented as the mean ± SD. Significant differences are indicated by **** *p* < 0.0001.

In the healthy colon cells, the reactivity to VGLL4 was undetectable in the epithelium, while it was strong in the lamina propria (Figure 3a). There were 31.90% VGLL4-positive cells detected in the healthy lamina propria (Figure 3f,g). Similar to the healthy tissues, reactivity to VGLL4 in Dukes A was detected only in the lamina propria (Figure 3b). There were 27.55% VGLL4-positive cells in the lamina propria of Dukes A (Figure 3f,g).

In the more advanced stage of CRC, Dukes B had very high reactivity to VGLL4 in the epithelium and, like the healthy colon and Dukes A, high reactivity in the lamina propria (Figure 3c). In Dukes B, there were 62.75% and 31.4% VGLL4-positive cells in the lamina propria and epithelium, respectively (Figure 3f,g). In Dukes C, expression of VGLL4 was detected in both the epithelium and lamina propria (Figure 3d). There were 8% VGLL4-positive cells in the epithelium and 40.7% in the lamina propria in Dukes C (Figure 3f,g). Interestingly, in the healthy colon and Dukes A, the VGLL4 expression pattern was more condensed around the nuclei, while in Dukes B and Dukes C, it was more cytoplasmic and dispersed (Figure 3a–d). The CRC stage Dukes D had almost undetectable levels of VGLL4 expression in both the epithelium and lamina propria (Figure 3e).

The expression analysis of VGLL4 in the epithelium between the control and CRC samples showed a significant increase in VGLL4-positive cells in Dukes B and Dukes C (*p* < 0.0001) (Figure 3f). Moreover, there were more VGLL4-positive cells in the epithelium of Dukes B compared to Dukes A, C, and D (*p* < 0.0001) (Figure 3f). In the epithelium of Dukes C, there were more VGLL4-positive cells compared to Dukes A and D (Figure 3f). In the lamina propria, a significant decrease in VGLL4-positive cells was detected between the healthy tissue and CRC stages Dukes C and Dukes D (*p* < 0.0001) (Figure 3g). A significant decrease in VGLL4-positive cells in Dukes C and Dukes D compared to Dukes A and B was detected (Figure 3g). Similarly, there was a significant decrease in VGLL4-positive cells between Dukes C and D (*p* < 0.0001) (Figure 3g).

The expression analysis of VGLL4 between the lamina propria and epithelium showed a significantly higher portion of VGLL4-positive cells in lamina propria compared to the epithelium in the healthy tissue and Dukes A (*p* < 0.0001) (Figure 3h). The opposite effect was detected in Dukes B and C. There was a significantly higher number of VGLL4-positive cells in the epithelium compared to the lamina propria (*p* < 0.0001) (Figure 3h). In Dukes D, VGLL4-positive cells were barely detected, so no statistical analysis could be performed (Figure 3e,h).

### 3.4. Immunofluorescence Staining with WNT4 Marker and Quantification of Immunoreactive Cells

**Figure 4:**
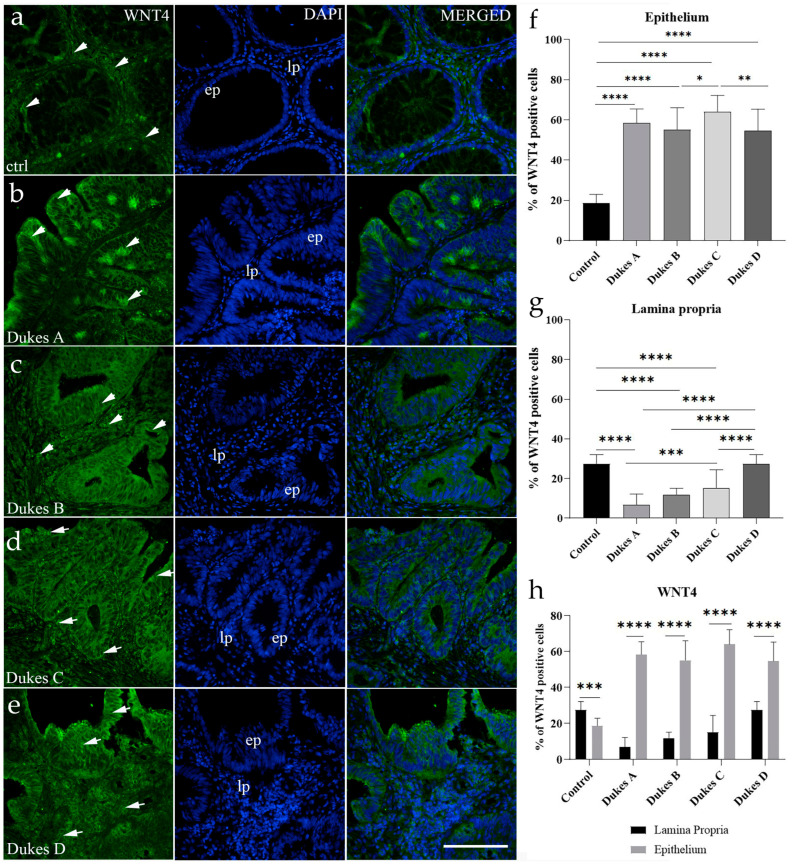
Immunofluorescence image of the control adult colon (**a**) and CRC stages Dukes A (**b**), Dukes B (**c**), Dukes C (**d**), and Dukes D (**e**). Arrows indicate positive WNT4 cells in the epithelium (ep) and lamina propria (lp). DAPI staining (blue nuclei). The percentages of cells positive to WNT4 staining in control and Dukes A–D CRC stages in the epithelium (**f**) and lamina propria (**g**) were calculated from a minimum of ten representative images. For (**a**–**e**), scale bar is 100 μm. For statistical analysis, one-way ANOVA followed by Tukey’s multiple comparison tests (**f**,**g**) and two-way ANOVA test and Šídák’s multiple comparisons test (**h**) were used. Data are shown as the mean ± SD. Significant differences are indicated by: * *p* < 0.05, ** *p* < 0.01, *** *p* < 0.001, **** *p* < 0.0001.

In the healthy control colon, reactivity to WNT4 was detected both in the epithelium and lamina propria (Figure 4a). There were 18.65% WNT4-positive cells detected in the healthy epithelium and 27.55% in the lamina propria (Figure 4f,g). In CRC Dukes A–D, reactivity to WNT4 was also detected in both areas, but with higher reactivity in the epithelium (Figure 4b–e). There were 58.40% WNT4-positive cells in the lamina propria and 6.90% in the epithelium of cancers at the Dukes A stage (Figure 4f,g). In samples staged as Dukes B, there were 55.15% and 11.8% WNT-positive cells in the epithelium and lamina propria, respectively (Figure 4f,g). The samples from CRC stage Dukes C had 64.10% WNT4-positive cells in the epithelium and 15.05% in the lamina propria (Figure 4f,g). In Dukes D, there were 54.7% WNT-positive cells in the epithelium and 27.55% in the lamina propria (Figure 4f,g).

The analysis of WNT4 protein expression in the epithelium between the control and CRC samples showed a significant increase in WNT-positive cells in CRC stages Dukes A–D (*p* < 0.0001) (Figure 4f). Moreover, there were more WNT4-positive cells in the epithelium of CRCs at Dukes C compared to those staged as Dukes B (*p* < 0.05) and D (*p* < 0.01) (Figure 4f). In the lamina propria, a significant decrease in WNT4-positive cells was detected between the healthy tissue and CRC stages Dukes A–C (*p* < 0.0001) (Figure 4g). Similarly, there was a significant decrease in WNT4-positive cells in Dukes A–C compared to CRCs staged as Dukes D (*p* < 0.0001) (Figure 4g). There was also a significant decrease in WNT4-positive cells between cancers staged as Dukes A and C (*p* < 0.001) (Figure 4g).

The analysis of WNT4 expression between the lamina propria and epithelium showed a significantly higher portion of WNT4-positive cells in the lamina propria of the healthy tissue compared to the epithelium (*p* < 0.001) (Figure 4h). The opposite was detected in Dukes A–D. There was a significantly higher percentage of WNT4-positive cells in the epithelium compared to the lamina propria in the CRC samples (*p* < 0.0001) (Figure 4h).

### 3.5. Differential Expression According to RNA Sequencing Data

**Figure 5:**
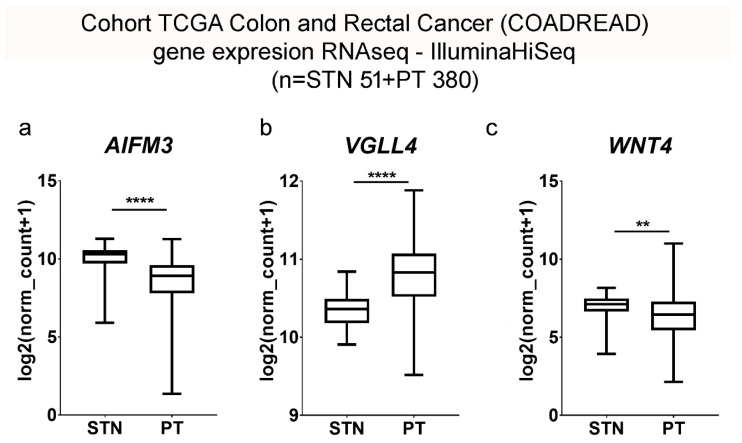
Cohort TCGA colon and rectal cancer (COADREAD) gene expression analysis of AIFM3 (**a**), VGLL4 (**b**), and WNT4 (**c**) by RNAseq–Illumina HiSeq between control (STN) and CRC patient groups (PT). Data are shown as log2(norm count + 1), with the use of unpaired *t*-test and Welch’s correction. Significant differences are indicated by: ** *p* < 0.01 and **** *p* < 0.0001.

RNA sequencing data from a publicly available COADREAD study was extracted to analyze the expression of the three genes of interest in CRC tumor and control tissue. Gene expression of AIFM3 (*p* < 0.0001), VGLL4 (*p* < 0.0001), and WNT4 (*p* < 0.01) was significantly altered in tumor tissues in comparison to the control tissues (Figure 5). AIFM3 and WNT4 genes had lower (Figure 5a,c) while VGLL4 had higher expression (Figure 5b) in CRC tumors compared to the control tissue.

### 3.6. Correlation Between Selected Markers and Patient Survival Analysis

**Figure 6:**
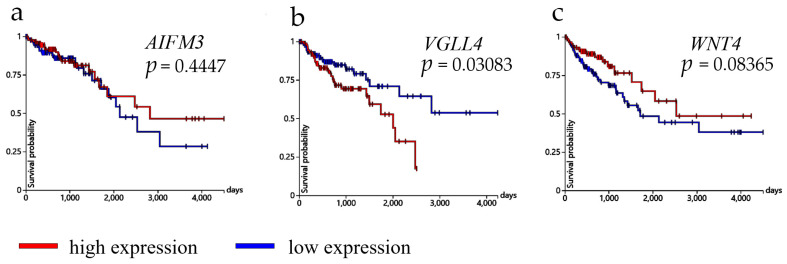
Graphic presentation of survival analysis (days) with expression of AIFM3 (**a**), VGLL4 (**b**), and WNT4 (**c**). mRNA expression of genes of interest in CRC patients was shown as the average time of survival in days. Red line represents high and blue line low expression. For statistical analysis, we used the Kaplan–Meier method with log-rank test for survival length. Data were obtained from the TCGA colon and rectal cancer (COADREAD) study.

The correlation between the survival rates and the level of mRNA expressions of AIFM3, VGLL4, and WNT4 in CRCs was analyzed (Figure 6). We observed no significant difference in survival in patients with CRC between the high- and low-expression groups with regard to AIFM3 (*p* = 0.447) and WNT4 (*p* = 0.08365) staining (Figure 6a,c). On the other hand, a statistically significant difference was observed in survival times (*p* = 0.03083) between the high- and low-expression groups with regard to VGLL4. A low expression of the VGLL4 displayed a correlation with regard to better survival probability (Figure 6b).

### 3.7. Expression Analysis of Observed Genes in Relation to Microsatellite Instability in Colorectal Cancer

**Figure 7:**
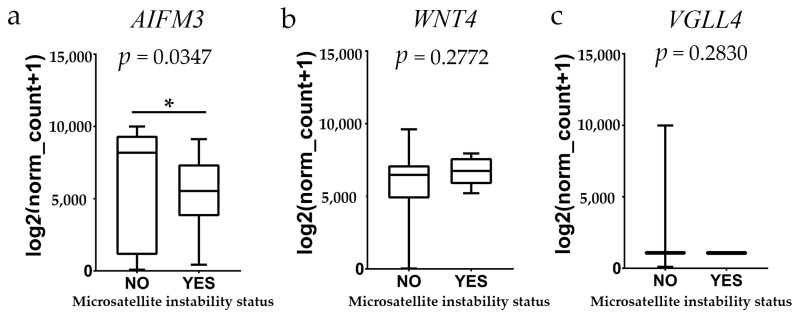
Expression analysis of AIFM3, WNT4, and VGLL4 genes in relation to microsatellite instability (MSI) status. The boxplots represent normalized log-transformed counts [log2(norm_count+1)] for samples with MSI status categorized as “NO” (microsatellite stable) or “YES” (microsatellite unstable). A significant difference in expression levels of AIFM3 (**a**) was observed between MSI-stable and MSI-unstable samples. No significant difference in expression levels of WNT4 (**b**) and VGLL4 (**c**) was observed. The horizontal line within each box represents the median, the box spans the interquartile range, and whiskers indicate the minimum and maximum values, excluding outliers. The data were analyzed using the Mann–Whitney U test. Significant difference is indicated by * *p* < 0.05.

The expression of AIFM3, WNT4, and VGLL4 in relation to microsatellite instability (MSI) status in colorectal cancer was analyzed using a cohort of 108 patients extracted from the TCGA colon and rectal cancer (COADREAD) study in the XENA database. Among the cohort, 98 patients were classified as MSI-stable, while 10 were MSI-unstable. Our results revealed a statistically significant difference in AIFM3 expression levels between MSI-stable and MSI-unstable groups (*p* = 0.0347), with higher expression observed in the MSI-stable group (Figure 7a). In contrast, WNT4 and VGLL4 expression did not significantly differ between the two groups (*p* = 0.2772 and *p* = 0.2830, respectively) (Figure 7b,c).

## 4. Discussion

Cancer development can be a consequence of hyperactivation of cellular proliferation and inadequate activation of apoptosis, both contributing to the uncontrolled survival of malignant cells [17]. Therefore, disruptions in the expression and function of signaling molecules involved in the regulation of apoptosis and cellular growth represent important molecular events involved in cancer initiation and progression. Stimulation of cancer cell death by activation of cellular apoptotic pathways and inhibition of uncontrolled proliferation are key targets in cancer therapies. The usual apoptotic markers, such as Fas receptor, Fas ligand, and circulating DNA, are often used to monitor therapy progress [18]. These are reasons for a continued search for novel—and investigation of known—molecules involved in the control of apoptosis and cellular proliferation, especially those that could serve as relevant prognostic markers for better survival and therapy targets. In this study, using tissue samples of patients with different stages of CRC, the expression levels of three important signaling molecular markers AIFM3, VGLL4, and WNT4 were evaluated.

Recently, apoptosis-related protein AIFM3 was found to be increased in breast cancer and cholangiocarcinoma [19]. Chua-on et al. reported elevated AIFM3 levels in the sera of cholangiocarcinoma patients, originating from the tumor tissue [20]. Unlike the data from breast cancer and cholangiocarcinoma patients, in our study, CRC patients’ samples showed a decrease in AIFM-positive cells in the lamina propria. Analysis revealed a significant reduction in AIFM-positive cells within the lamina propria, and this reduction was mostly pronounced in advanced CRC stages (Dukes B–D), where AIFM-positive cells were very low or undetectable. Furthermore, the epithelial regions of CRC samples exhibited a markedly low percentage of AIFM-positive cells. These findings suggest that the loss of AIFM-positive cells may be associated with CRC progression and highlight a potential divergence in the tumor microenvironment between CRC and other cancer types, indicating possible tissue specificity. To elucidate the functional consequences of reduced AIFM-positive cells’ presence in CRCs, further studies are warranted.

Another important hallmark of tumors is uncontrollable cellular growth. This usually occurs through mutations in cellular checkpoint proteins, dysregulation of tumor suppressor genes such as p53, and activation of oncogenes or mutations in components of growth signaling receptors and downstream pathways, leading to their activation and increased cellular proliferation [21]. Two very important pathways for cellular growth are the Hippo–YAP1 and Wnt/β-catenin pathways. The activity of these pathways can be used as a diagnostic marker in cancer prognosis, and high activation correlates with worse survival rates [22]. One of the described prognostic markers is VGLL4 [22]. In breast cancer, it binds TEAD1, and this interaction antagonizes TEAD1–YAP1-induced cell proliferation in Hippo–YAP1 signaling. In CRCs, it was shown that the binding of VGLL4 to the TEAD4–TCF4 complex inhibits Wnt/β-catenin signaling [23]. Our findings support the growing evidence that low expression of VGLL4 is associated with poorer survival outcomes in various cancers, particularly breast and colorectal cancer [23,24]. In breast cancer, diminished VGLL4 levels correlate with shorter relapse-free and disease-specific survival, highlighting its role as an independent prognostic factor [24]. This aligns with studies demonstrating that VGLL4 overexpression inhibits cancer cell proliferation and migration, suggesting its function as a tumor suppressor [23,24].

Similarly, in colorectal cancer, reduced VGLL4 expression is linked to worse survival and inversely associated with Wnt/β-catenin target gene expression. Lower levels of VGLL4 also correlate with adverse clinical parameters such as tumor size and lymph node metastasis. Our results here using CRC samples partially confirmed these data. A decrease in VGLL4-positive cells was detected in the lamina propria in later stages of CRC at Dukes C and D. However, the results differed in the epithelium—no VGLL4-positive cells could be detected in healthy and early-stage CRC Dukes A. One can argue that in early tumor stages under normal conditions, VGLL4 activity is not needed or is limited; however, with the tumor progression beyond mucosa, as in cancers of the Dukes B stage, it is upregulated and serves as a tumor suppressor. Our findings reinforce VGLL4’s importance in CRC progression and indicate a need for further investigation into its mechanistic role as a tumor suppressor, especially in CRC cancers.

The third protein investigated in this study was WNT4, a protein likely to be involved in the progression of CRC, and its expression might serve as an important progression indicator. Activators of the Wnt pathway, such as WNT4, can be classified as tumor promoters [25]. Canonical Wnt signaling, also known as Wnt/β-catenin, relies on nuclear translocation of β-catenin and activation of downstream genes regulating cell proliferation. Generally, turnover of β-catenin is regulated by phosphorylation-dependent ubiquitination by a complex formed by glycogen synthase kinase 3β (GSK3β), casein kinase I (CK I), Axin, and adenomatous polyposis (APC). Once the Wnt ligand binds to its core receptor (LRP5/6 and frizzled proteins), it disrupts the GSK3β/CK I/Axin/APC complex by recruiting activated cytosolic Dvl protein. This leads to β-catenin cytosolic accumulation and, finally, translocation to the nucleus. There, β-catenin works with other transcription factors to activate Wnt target genes [26]. The WNT4 protein activates canonical Wnt signaling and epithelial–mesenchymal transition (EMT) in CRC. It is found in elevated levels in the serum of CRC patients [15]. Results in the study here showed the upregulated expression of WNT4 in the epithelium of CRC samples at Dukes A–D. However, the percentage of WNT-positive cells in the lamina propria was seen to increase from the early-stage to late-stage Dukes A to Dukes D. This could be a manifestation of epithelial–mesenchymal transition (EMT) initiation, which happens in more advanced stages of CRC, Dukes C and D. This represents excellent pilot data that could be considered in future studies focused on the evaluation of WNT4’s role in CRC.

Microsatellite instability (MSI) represents a significant form of genetic hypermutability stemming from deficiencies in the DNA mismatch repair (MMR) system. This phenomenon is particularly relevant in colorectal cancer (CRC), where MSI is detected in approximately 15–20% of cases, predominantly as a result of sporadic hypermethylation of the MLH1 promoter or associated with Lynch syndrome due to germline mutations in MMR genes such as MLH1, MSH2, MSH6, and PMS [27,28]. The presence of MSI in tumors is characterized by distinct molecular features, including a higher tumor mutational burden and an increased neoantigen load, which correlate with enhanced immune responses [29,30]. Clinically, MSI-unstable tumors are often associated with better prognoses and improved responses to immune checkpoint inhibitors compared to their microsatellite-stable (MSS) counterparts [31]. As such, MSI status serves as a critical biomarker for diagnosis, prognosis, and therapeutic decision-making in CRC management, highlighting its essential role in tailoring patient-specific treatment strategies [28,32].

In this study, we conducted a comprehensive analysis of microsatellite instability (MSI) in colorectal cancer (COADREAD), utilizing data sourced from the XENA database. Our analysis included a cohort of 108 patients, among which 98 were classified as MSI-stable and 10 as MSI-unstable. Gene expression profiling was performed to identify potential biomarkers associated with MSI status. Notably, AIFM3 exhibited a statistically significant difference in expression levels between the MSI-stable and MSI-unstable groups, suggesting its potential utility as a biomarker for MSI in colorectal cancer. In contrast, VGLL4 and WNT4 did not show significant differences in expression between the two groups, indicating that these genes may not serve as reliable indicators of MSI status in this context. These findings contribute to the understanding of the molecular underpinnings of colorectal cancer and highlight AIFM3 as a candidate biomarker warranting further investigation.

The primary limitation of our study is the relatively small sample size (N = 43), which may impact the generalizability of the findings. A larger cohort would provide more robust statistical power and strengthen the validity of the conclusions. Additionally, while this study focused on specific proteins, further exploring other proteins and genes within the Wnt signaling pathway could offer a more comprehensive understanding of their roles in CRC. These limitations highlight areas for future research, which we aim to pursue as part of a broader investigation.

## 5. Conclusions

This study provides novel findings highlighting the expression levels of three important apoptotic and growth signaling proteins, AIFM3, VGLL4, and WNT4, involved in different clinically relevant stages of progression in patients with colorectal cancer (CRC). Data also provide an excellent basis for future, more in-depth studies on molecular mechanisms in CRC associated with the expression and function of these proteins and related signaling pathways.

The key findings involve a significant reduction in AIFM3-positive cells in the lamina propria, especially in advanced CRC stages (Dukes B–D), contrasting with increased AIFM3 levels in breast cancer and cholangiocarcinoma. Furthermore, AIFM3 showed a statistically significant difference in expression between MSI-stable and MSI-unstable colorectal cancer, highlighting its potential as a biomarker for MSI status. VGLL4, a known prognostic marker and tumor suppressor, showed decreased expression in the lamina propria during late CRC stages, suggesting that its downregulation correlates with the tumor progression beyond mucosa. Conversely, WNT4, a Wnt pathway activator, exhibited increasing expression in both the epithelium and lamina propria across CRC stages, consistent with its role in epithelial–mesenchymal transition (ETM) in advanced disease.

Therefore, this study highlights the clinical potential of AIFM3, VGLL4, and WNT4 in colorectal cancer (CRC) progression. VGLL4 emerges as a promising prognostic biomarker, with its decreased expression correlating with advanced disease stages. Similarly, the upregulation of WNT4, a key mediator of epithelial–mesenchymal transition, suggests its role in promoting metastasis and its potential as a therapeutic target, while the reduction of AIFM3-positive cells in CRC compared to other cancers highlights its potential role in restoring apoptotic pathways. The distinct expression patterns of these proteins in the tumor epithelium and the surrounding microenvironment underscore their importance in shaping tumor progression and providing a foundation for personalized diagnostic and therapeutic strategies. Future studies will focus on elucidating the mechanisms underlying these findings and validating their clinical utility in CRC management. 

## Figures and Tables

**Table 1:** Patients’ characteristics according to CRC stages.

Histology and Stage		No. of Patients (n)	Sex (n)	Age (Year)
CRC	Dukes A	12	M (6) F (6)	56.83 ± 6.15 51.83 ± 5.87
	Dukes B	11	M (5) F (6)	75.8 ± 4.07 82.17 ± 7.77
	Dukes C	10	M (8) F (2)	69 ± 9.46 69 ± 7.07
	Dukes D	10	M (7) F (3)	66.29 ± 10.20 61.33 ± 7.72

CRC—colorectal cancer; M—male; F—female. Values are means ± standard deviations.

**Table 2:** Primary and secondary antibodies used for immunohistochemistry.

Primary Antibody/Dilution	Secondary Antibody/Dilution
VGLL4 rabbit polyclonal antibody, Invitrogen (PA5-58467)/1:50	Alexa Fluor 488-conjugate AffiniPure DonkeyAnti-Rabbit IgG(H + L), Jackson Laboratories (711-545-152)/1:300
Wnt 4, rabbit polyclonal antibody, Abcam (ab91226)/1:20	Alexa Fluor 488-conjugate AffiniPure DonkeyAnti-Rabbit IgG(H + L), Jackson Laboratories (711-545-152)/1:300
AIFM3, rabbit polyclonal antibody, OriGene Technologies, Inc. (Rockville, MD, USA) (OG-AP22615PU-N)/1:400	Alexa Fluor 488-conjugate AffiniPure DonkeyAnti-Rabbit IgG(H + L), Jackson Laboratories (711-545-152)/1:300

## Data Availability

The raw data supporting the conclusions of this article will be made available by the authors upon written request.

## Supplementary Materials

The following supporting information can be downloaded at: https://www.mdpi.com/article/10.3390/cancers17020166/s1, Figure S1: Negative control staining images for colorectal cancer tissues at Dukes’ C stage, assessing AIFM3 (a), VGLL4 (b) and WNT4 (c).

## References

[1] Prager G.W., Braga S., Bystricky B., Qvortrup C., Criscitiello C., Esin E., Sonke G.S., Martinez G.A., Frenel J.S., Karamouzis M. (2018). Global cancer control: Responding to the growing burden, rising costs and inequalities in access. ESMO Open.

[2] Fadlallah H., El Masri J., Fakhereddine H., Youssef J., Chemaly C., Doughan S., Abou-Kheir W. (2024). Colorectal cancer: Recent advances in management and treatment. World J. Clin. Oncol..

[3] Lewandowska A., Rudzki G., Lewandowski T., Stryjkowska-Gora A., Rudzki S. (2022). Risk Factors for the Diagnosis of Colorectal Cancer. Cancer Control J. Moffitt Cancer Cent..

[4] Goenka A., Khan F., Verma B., Sinha P., Dmello C.C., Jogalekar M.P., Gangadaran P., Ahn B.C. (2023). Tumor microenvironment signaling and therapeutics in cancer progression. Cancer Commun..

[5] Mukai S., Hirata Y., Ishikawa S., Kai A., Kohata A., Okimoto S., Fujisaki S., Fukuda S., Takahashi M., Fukuda T. (2020). Mixed neuroendocrine-non-neuroendocrine neoplasms of ascending colon: A case report. Int. J. Surg. Case Rep..

[6] Hong Y., Kim J., Choi Y.J., Kang J.G. (2020). Clinical study of colorectal cancer operation: Survival analysis. Korean J. Clin. Oncol..

[7] Hossain M.S., Karuniawati H., Jairoun A.A., Urbi Z., Ooi J., John A., Lim Y.C., Kibria K.M.K., Mohiuddin A.K.M., Ming L.C. (2022). Colorectal Cancer: A Review of Carcinogenesis, Global Epidemiology, Current Challenges, Risk Factors, Preventive and Treatment Strategies. Cancers.

[8] Akkoca A.N., Yanik S., Ozdemir Z.T., Cihan F.G., Sayar S., Cincin T.G., Cam A., Ozer C. (2014). TNM and Modified Dukes staging along with the demographic characteristics of patients with colorectal carcinoma. Int. J. Clin. Exp. Med..

[9] Muller M.F., Ibrahim A.E., Arends M.J. (2016). Molecular pathological classification of colorectal cancer. Virchows Arch. Int. J. Pathol..

[10] Dunne P.D., Arends M.J. (2024). Molecular pathological classification of colorectal cancer—An update. Virchows Arch. Int. J. Pathol..

[11] Xie Q., Lin T., Zhang Y., Zheng J., Bonanno J.A. (2005). Molecular cloning and characterization of a human AIF-like gene with ability to induce apoptosis. J. Biol. Chem..

[12] Zheng A., Zhang L., Song X., Wang Y., Wei M., Jin F. (2019). Clinical implications of a novel prognostic factor AIFM3 in breast cancer patients. BMC Cancer.

[13] Deng X., Fang L. (2018). VGLL4 is a transcriptional cofactor acting as a novel tumor suppressor via interacting with TEADs. Am. J. Cancer Res..

[14] Zhang Q., Pan Y., Ji J., Xu Y., Qin L. (2021). Roles and action mechanisms of WNT4 in cell differentiation and human diseases: A review. Cell Death Discov..

[15] Yang D., Li Q., Shang R., Yao L., Wu L., Zhang M., Zhang L., Xu M., Lu Z., Zhou J. (2020). WNT4 secreted by tumor tissues promotes tumor progression in colorectal cancer by activation of the Wnt/beta-catenin signalling pathway. J. Exp. Clin. Cancer Res..

[16] Vukojevic K., Filipovic N., Tica Sedlar I., Restovic I., Bocina I., Pintaric I., Saraga-Babic M. (2016). Neuronal differentiation in the developing human spinal ganglia. Anat. Rec..

[17] Wong R.S. (2011). Apoptosis in cancer: From pathogenesis to treatment. J. Exp. Clin. Cancer Res..

[18] Holdenrieder S., Stieber P. (2004). Apoptotic markers in cancer. Clin. Biochem..

[19] Chua-On D., Proungvitaya T., Techasen A., Limpaiboon T., Roytrakul S., Tummanatsakun D., Araki N., Proungvitaya S. (2022). Bioinformatic Prediction of Novel Signaling Pathways of Apoptosis-inducing Factor, Mitochondrion-associated 3 (AIFM3) and Their Roles in Metastasis of Cholangiocarcinoma Cells. Cancer Genom. Proteom..

[20] Chua-On D., Proungvitaya T., Tummanatsakun D., Techasen A., Limpaiboon T., Roytrakul S., Wongkham S., Wongkham C., Somintara O., Sangkhamanon S. (2020). Apoptosis-Inducing Factor, Mitochondrion-Associated 3 (AIFM3) Protein Level in the Sera as a Prognostic Marker of Cholangiocarcinoma Patients. Biomolecules.

[21] Wang H., Guo M., Wei H., Chen Y. (2023). Targeting p53 pathways: Mechanisms, structures, and advances in therapy. Signal Transduct. Target. Ther..

[22] Liu J., Xiao Q., Xiao J., Niu C., Li Y., Zhang X., Zhou Z., Shu G., Yin G. (2022). Wnt/beta-catenin signalling: Function, biological mechanisms, and therapeutic opportunities. Signal Transduct. Target. Ther..

[23] Jiao S., Li C., Hao Q., Miao H., Zhang L., Li L., Zhou Z. (2017). VGLL4 targets a TCF4-TEAD4 complex to coregulate Wnt and Hippo signalling in colorectal cancer. Nat. Commun..

[24] Kim J.Y., Kim E.K., Lee W.M., Hong Y.O., Lee H. (2020). VGLL4 with low YAP expression is associated with favorable prognosis in colorectal cancer. APMIS Acta Pathol. Microbiol. Immunol. Scand..

[25] Sun L., Xing J., Zhou X., Song X., Gao S. (2024). Wnt/β-catenin signalling, epithelial-mesenchymal transition and crosslink signalling in colorectal cancer cells. Biomed. Pharmacother..

[26] Zhao H., Ming T., Tang S., Ren S., Yang H., Liu M., Tao Q., Xu H. (2022). Wnt signaling in colorectal cancer: Pathogenic role and therapeutic target. Mol. Cancer.

[27] Boland C.R., Goel A. (2010). Microsatellite instability in colorectal cancer. Gastroenterology.

[28] Nojadeh J.N., Behrouz Sharif S., Sakhinia E. (2018). Microsatellite instability in colorectal cancer. Excli J..

[29] Nádorvári M.L., Lotz G., Kulka J., Kiss A., Tímár J. (2024). Microsatellite instability and mismatch repair protein deficiency: Equal predictive markers?. Pathol. Oncol. Res..

[30] Vilar E., Gruber S.B. (2010). Microsatellite instability in colorectal cancer—The stable evidence. Nat. Rev. Clin. Oncol..

[31] Buckowitz A., Knaebel H.P., Benner A., Bläker H., Gebert J., Kienle P., von Knebel Doeberitz M., Kloor M. (2005). Microsatellite instability in colorectal cancer is associated with local lymphocyte infiltration and low frequency of distant metastases. Br. J. Cancer.

[32] Söreide K., Janssen E.A.M., Söiland H., Körner H., Baak J.P.A. (2006). Microsatellite instability in colorectal cancer. Br. J. Surg..

