# Supplementary material for: Expression Pattern of AIFM3, VGLL4, and WNT4 in Patients with Different Stages of Colorectal Cancer
Source: Cancers (Basel). 2025 Jan 7;17(2):166. doi: 10.3390/cancers17020166 (PMC11763972; doi:10.3390/cancers17020166)
Supplement: Supplementary file 1 [file cancers-17-00166-s001.zip › cancers-3366172-supplementary.docx]

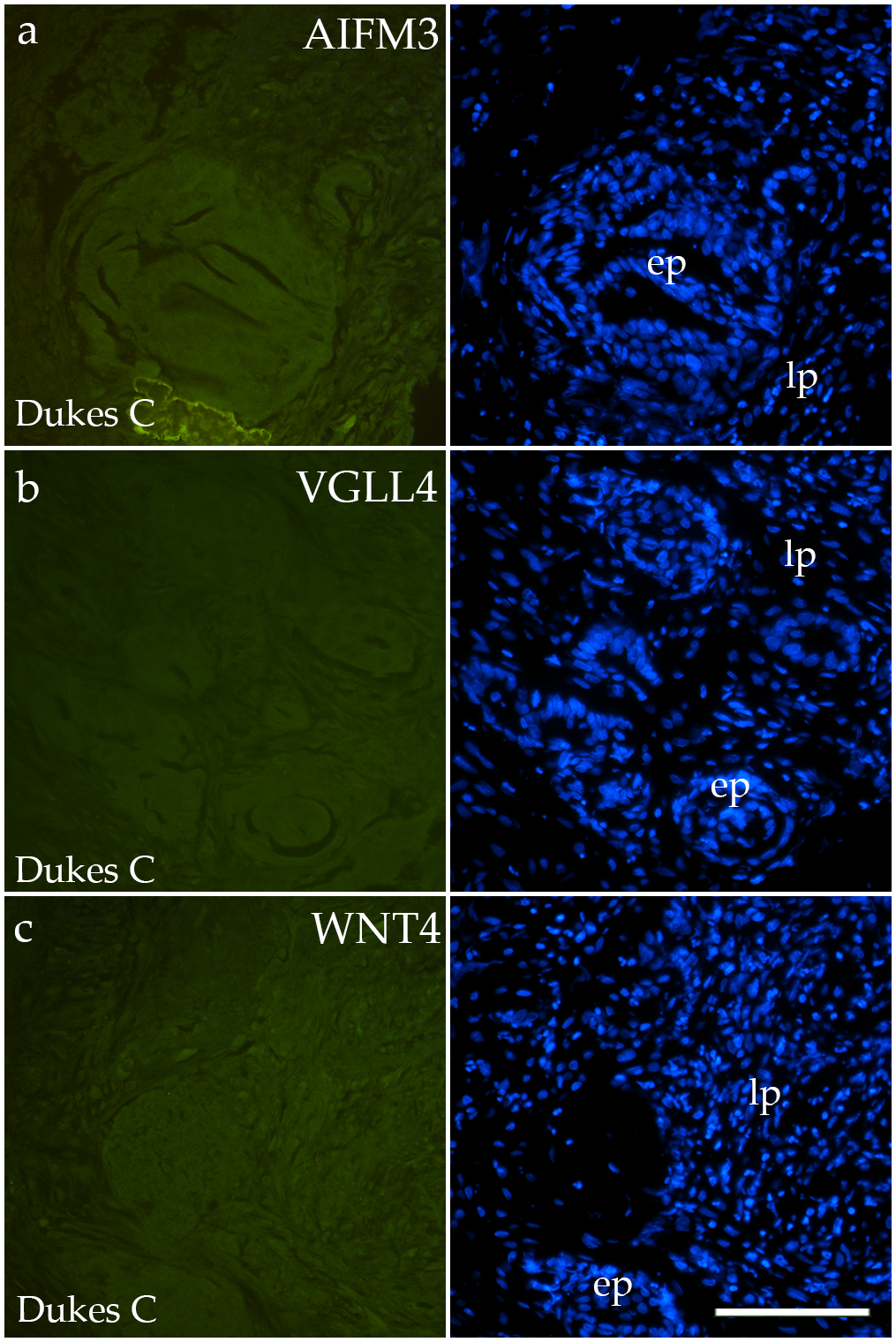


**Figure S1.** Negative control staining images (primary antibodies were omitted from the immunofluorescent protocol, and only secondary antibodies were applied) for colorectal cancer tissues at Dukes’ C stage, assessing AIFM3 (a), VGLL4 (b) and WNT4 (c). Images were taken at ×40 magnification, with a scale bar of 100 μm applied to all images.
